# hPSCreg—the human pluripotent stem cell registry

**DOI:** 10.1093/nar/gkv963

**Published:** 2015-09-22

**Authors:** Stefanie Seltmann, Fritz Lekschas, Robert Müller, Harald Stachelscheid, Marie-Sophie Bittner, Weiping Zhang, Luam Kidane, Anna Seriola, Anna Veiga, Glyn Stacey, Andreas Kurtz

**Affiliations:** 1 Berlin-Brandenburg Center for Regenerative Therapies, Charité University Medicine Berlin, Berlin, 13353, Germany; 2 Berlin Institute of Health—Stem Cell Core Facility, 13353 Berlin, Germany; 3 National Institute for Biological Standards and Control, South Mimms EN63QG, UK; 4 Center of Regenerative Medicine in Barcelona, Barcelona Stem Cell Bank, Barcelona 08003, Spain; 5 Seoul National University, College of Veterinary Medicine and Research Institute for Veterinary Science, Seoul 151-742, Republic of Korea

## Abstract

The human pluripotent stem cell registry (hPSCreg), accessible at http://hpscreg.eu, is a public registry and data portal for human embryonic and induced pluripotent stem cell lines (hESC and hiPSC). Since their first isolation the number of hESC lines has steadily increased to over 3000 and new iPSC lines are generated in a rapidly growing number of laboratories as a result of their potentially broad applicability in biomedicine and drug testing. Many of these lines are deposited in stem cell banks, which are globally established to store tens of thousands of lines from healthy and diseased donors. The Registry provides comprehensive and standardized biological and legal information as well as tools to search and compare information from multiple hPSC sources and hence addresses a translational research need. To facilitate unambiguous identification over different resources, hPSCreg automatically creates a unique standardized name for each cell line registered. In addition to biological information, hPSCreg stores extensive data about ethical standards regarding cell sourcing and conditions for application and privacy protection. hPSCreg is the first global registry that holds both, manually validated scientific and ethical information on hPSC lines, and provides access by means of a user-friendly, mobile-ready web application.

## INTRODUCTION

The research landscape for human pluripotent stem cells (hPSC) is changing rapidly. The establishment of human embryonic stem cells (hESC) in 1998 (1) and human induced pluripotent stem cells (hiPSC) in 2007 (2) has provided new tools for cell biology, regenerative medicine, disease modeling and drug and toxicity testing. The establishment of thousands of human ESC and iPSC lines, deposited in multiple national and international cell banks, aims to match the growing demands from these fields. Together with the increasing demand for hPSC lines, technologies for their characterization and modification, including omics, functional cell assays and tools for genetic modification, are constantly being improved. At the same time, higher standards for characterization have to be fulfilled. A registry for human PSC needs to provide an inventory of available cell lines throughout the diverse resources together with validated characterization data and information on cell line origin and application. Since applicability depends on the donor consent, e.g. regarding access to genetic data and commercial use, information on the ethical and regulatory environment under which these cells were obtained is highly relevant.

The Human Pluripotent Stem Cell Registry (hPSCreg; http://hpscreg.eu), established in 2007 with funding from the European Commission, originally aimed to provide transparency and comparability as well as management of ethical compliance in the dynamic yet controversial field of human ESC research (3,4). With the establishment of human iPSC, the registry was expanded and the database and user interface completely rebuilt to allow for registration of a broad set of human PSC-line related data. Where possible, the use of ontology and other standard terms was implemented to annotate lines. Importantly, registration is accompanied by automatic assignment of a unique name for each hPSC line, based on a standardized nomenclature. Availability of lines and regulatory background for their use is visualized in an interactive world map. All information is validated before publication, following a standardized internal process.

Currently there are 759 cell lines from 25 different countries registered in hPSCreg, thereof 683 hESC and 76 hiPSC lines. hPSCreg registers hPSC lines of existing cell banks and registries, including the European Bank of induced pluripotent Stem Cells (EBiSC), the Human iPSC Initiative (HipSci), WiCell Research Institute, the Korean Stem Cell registry and NIMH stem cell center at Rutgers. An exemplary pipeline established with EBiSC requires registration of an hiPSC line in hPSCreg, which assigns a name together with a BioSample ID (https://www.ebi.ac.uk/biosamples/, (5)) via the European Bioinformatics Institute (EMBL-EBI) to facilitate immediate identification and data access by EBiSC with unique identifiers from donor to cell line, batch or lot level.

## CELL DATA REGISTRATION AND MANAGEMENT

### Registration

**Figure 1.**
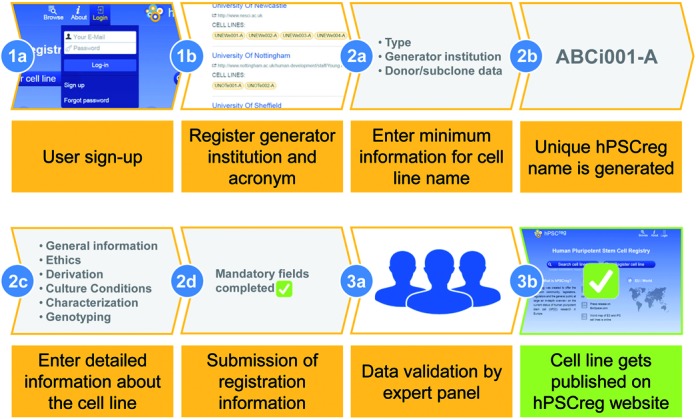
Registration process for a hPSC line. First, to access the cell line registration, users have to sign-up for an account (1a). A valid generator institution with a unique two to five letter acronym has to be registered or chosen from a list of already registered institutions (1b). To register a single cell line, users need to enter the minimum data (2a), allowing the creation of a unique hPSCreg name (2b) according to the hPSCreg nomenclature. Following, detailed cell line information (2c) has to be entered. To finally submit, a set of 52 mandatory fields have to be completed (2d). An international expert panel will validate the given information about the submitted cell line (3a). After successful validation, the cell line is published publicly on hPSCreg's website (3b).

Data acquisition is achieved by user-initiated registration of a cell line (Figure 1). hPSCreg developed and implemented an online registration tool of more than 740 possible data fields, which allows in-depth provision of information on each registered cell line (Table 1). The data fields were identified in close collaboration with the generators and users of human PSC lines. Registration information includes data about the provider of the data, characteristics of the donor of the tissue or cells used to generate hPSC-lines, the methods used to derive a cell line from the donated tissues, the cultivation conditions used for the hPSC-line and details on their phenotype and genotype. Donor related data includes information on the consenting process used for tissue donation and the content of the consented data use.

**Table 1.** Data fields available for hESC and hiPSC registration

	Number of fields	
Cell type	hiPSC	hESC
**Provider information**	**41**	**41**
*Providers/Generators*		
*Depositors*		
*Owners*		
*Distributors*		
**Donor information**	**82**	**66**
*General information (age, sex, ethnicity)*		
*Phenotype/Disease associated information*		
*Genetic background*		
*Ethical information*		
**Cell line information**	**625**	**607**
*General cell line information*		
*Derivation*		
*Culture conditions*		
***Characterization***	***460***	***460***
*Marker expression*		
*Differentiation potency*		
*QC documentation*		
***Genotype***	***91***	***91***
*Mutations*		
*STR, HLA*		
*Genetic modification*		
Application feedback		
***Publications***	***1***	***1***
*Projects*	*na*	*na*
Total	749	715

The online registration tool provides more than 740 possible data fields for hPSC, which allows in-depth provision of detailed information on each registered cell line.

The registration is divided into three levels of data provision (Table 2), which contain (i) mandatory information to automatically assign a unique name to the line, (ii) mandatory data on a minimal set of information to provide evidence that a line is pluripotent and has been derived within an appropriate ethical framework and (iii) non-mandatory information beyond the minimal dataset to refine and expand donor and cell line specific information, for example with genotyping data.

**Table 2.** Mandatory fields for the nomenclature and minimal data for registration of an hPSC line in hPSCreg

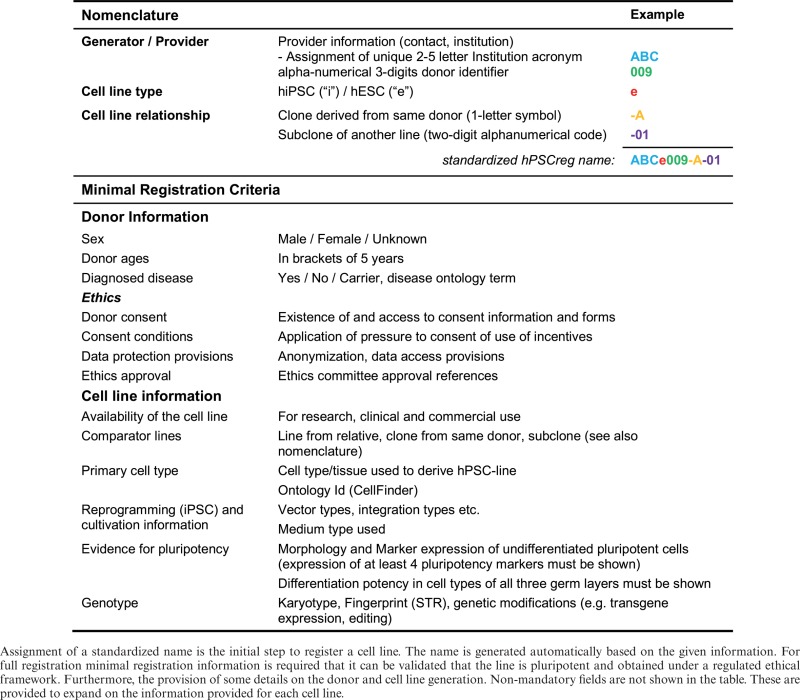		

Information required for applying a standardized nomenclature to automatically assign a unique name and identifier for each cell line is based on the system proposed by Luong *et* *al*. (6), with modifications (Table 2). It contains a unique acronym to identify the provider institute, a cell type descriptor such as ‘e’ for hESC and ‘i’ for hiPSC, a unique alphanumerical donor name and information on the relatedness of a cell line to a specific donor, e.g. whether the cell line is another clone from the same donor or a cell line is a subclone of another line. The implemented nomenclature allows in theory the assignment of 12 356 604 cell line generators to name 46 655 different donors, for each donor 26 different cell lines, for each cell line 1330 subclones and hence can provide more than 3 billion cell lines per provider. The Registry provides an application program interface (API) at http://hpscreg.eu/api/create_name to programmatically generate new hPSCreg cell line names, which is especially important for companies and research project that generate large numbers of new hPSC lines, which can be named with a standard hPSCreg-name while they are being generated. A detailed documentation about this functionality is available at http://hpscreg.eu/about/naming-tool.

Minimal information is mandatory to assess whether a cell line is pluripotent (Table 2). It requires evidence that at least four generally accepted pluripotency marker genes are expressed and that the cells can differentiate into cells of each of the three germ layers (endoderm, ectoderm and mesoderm). In addition, minimal information requires evidence that a prudent and ethical process was followed for the consenting of the embryo or tissue donors. The required information is naturally different between human ESC and iPSC lines, but generally asks for information on ethics approvals, the consenting process and the provisions provided in the consent form signed by the donor. Finally, the provider must inform about applicable restrictions for the use of the cell line for research. Altogether, up to 52 fields are mandatory for registration of a line.

Additional registration information includes phenotype and genotype information of the tissue donors, derivation and cultivation details and genotype information of the cell line. These data fields are not mandatory, but provide information important for potential users.

Where applicable, terms from existing ontologies are used this includes for example descriptions of diseases, cell types and tissues. To easily find the right ontology term, the correlating fields in the online registration form are linked to CellFinder (http://www.cellfinder.org, (7)), where users can search terms across a broad range of ontologies and retrieve ontology terms (8) alongside detailed descriptions to foster confidence in term selection. To aid data quality and ease of registration, hPSCreg implemented few literal text inputs and strongly relies on predefined field values. Experimental protocols and images can be uploaded to provide even richer descriptions. Omics datasets (e.g. from cDNA microarrays, RNA-Seq, CHiP-Seq) deposited elsewhere are linked to cell lines using the appropriate database identifier (e.g. BioSample ID, GEO ID).

To reduce uncertainty and accidental errors during registration, hPSCreg provides extensive help texts for each input.

### Validation

The quality of data available on hPSCreg and its ethical acceptability is of utmost importance. We have established strict criteria to ensure high quality through an open, fair and scientifically rigorous process. This process puts a strong focus on standardization of data input to streamline comparison and validation of data on cell lines from different sources.

To control data accuracy, hPSCreg uses a three-step registration system (Figure 1). First, prior to cell line registration, the user has to sign up for an account in order to supply contact details. This is crucial in order to provide full provenance and to allow communication between data curators and users. After hPSCreg has verified that the applicant is *bona* *fide* provider (i.e. from a recognized research organization) the account will be validated. In the second step, the approved users enter cell line related information. After at least the mandatory nomenclature and minimal information has been provided, the provided information gets manually checked by the hPSCreg data curators. In case of incomplete information or plausibility issues, the provider is contacted for clarification. In the last validation step, the hPSCreg Committee of National Representatives (CNR) is informed and can provide additional feedback on the validity of the data. Only cell lines validated by the hPSCreg data curators and the CNR will be made available to the public.

Additional validation can be provided by feedback from users. While this is in principle possible already by editing information provided for a cell line in hPSCreg's registration tool followed by manual validation, the feedback process will be expanded and refined in future versions of the Registry.

### Access

To ease data access, retrieval and understanding, hPSCreg follows a user-centered design approach by implementing an easy-to-use, mobile-friendly and aesthetically pleasing web application. The application's goal is to help users find cell lines and related data as quickly as possible. The interface design is kept at a minimum with focus on content, offering three ways of data discovery: searching, browsing and visual exploration.

Text-based searching is designed to be simple but powerful and is omnipresent in hPSCreg, i.e. a search can be started from any page. Search results are ranked according to their relevance by default but can be sorted by name or date as well. The result list shows the hPSCreg name alongside a list of alternative cell line identifiers, the contact institution, the derivation country and date, diseases associated with the specific cell line. Furthermore, the interface implements instantaneous faceted search to limit the search space to cell type, countries, diseases and derivation date.

In terms of browsing, hPSCreg provides the possibility to access all available cell lines via facet filter enabled lists just like the search results. Furthermore, cell line provider and hPSC research projects can be browsed through searchable, sortable and filter-enabled lists. For example, providers can be narrowed down by role, i.e. cell line generator, owner or distributor. As projects in which a particular cell line is used can be registered, and all EU-funded projects using hPSC must be registered at hPSCreg (9), these can be sorted alphabetically as well as by start- or end-date of the project. Lastly, hPSCreg offers a list of countries with information regarding registered cell lines, research projects and legal information.

**Figure 2.**
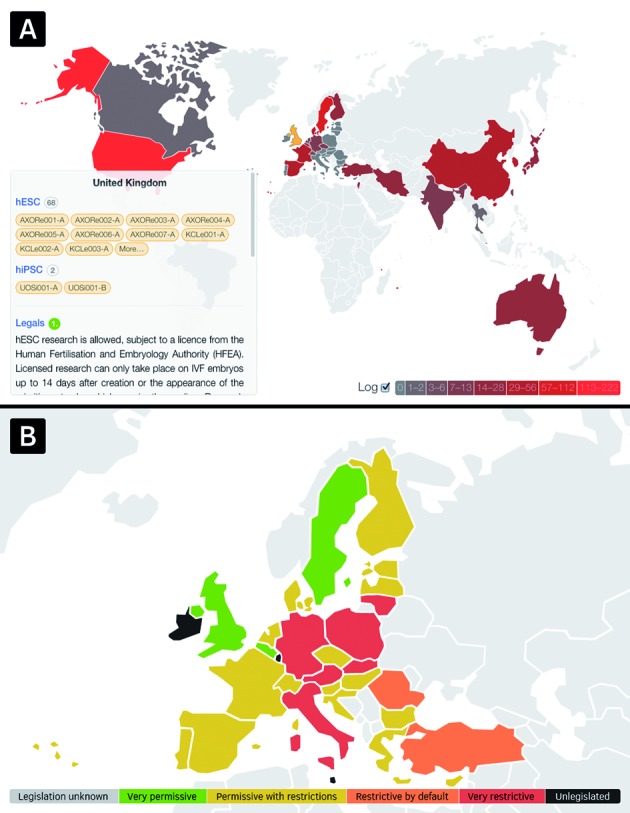
Interactive world map. Exploration of the worldwide distribution of registered cell lines and country-wise legalization: (**A**) shows the world wide log-transformed distribution of registered hPSC lines in hPSCreg. Colors range from dark gray (least cell lines registered) to saturated red (most cell lines registered). Countries without any registered cell line are colored in light gray. Note that in the printed version of this article colors are shown in gray scale. (**B**) Shows a zoomed view of Europe, displaying legal information by a five-grade color system. The coloring is an approximation of the exact legal state. More details are available on the country specific pages which are linked to the map.

To ease summarizing of available data in hPSCreg and the current state of hESC and hiPSC research around the world, an interactive colored world map has been implemented (Figure 2), visualizing the absolute and log-transformed distribution of registered hESC and hiPSC lines as well as the current state of ES cell research related legislation per country. The world map is draggable and zoomable and shows country-specific information upon selection by a mouse-click or finger-tab, which is cross-linked to detailed pages.

To improve hPSC line identification and minimize ambiguities, a simple yet semantically meaningful system for information retrieval is needed. A semantic uniform resource locator (URL) for every registered and validated cell line is provided by hPSCreg. Such a persistent URL will not change over time, allowing their use as uniform resource identifiers (URI) in future application ontologies. Semantic URLs in hPSCreg follow the schema: <ORGANIZATION>/<RESOURCE>/<IDENTIFIER>. Hereby, the organization and resource will be http://hpscreg.eu and cell-line respectively, while the identifier is a standardized hPSCreg name. For example, qualitative meta information of the hESC line WAe009-A (hPSCreg-derived standard name; also called WA09 or H9) can be accessed at http://hpscreg.eu/cell-line/WAe009-A.

Access to certain information falls under a managed access code, based on the consent provided by the original tissue donor to protect privacy. This includes genetic information and some clinical data, which may be used to identify the donor (10). Managed access for genetic data is only possible through data access committees, which are entitled to permit access to cell-line specific data deposited in central databases such as the European Genome-phenome Archive (EGA). Genetic data that are not under a managed access policy are directly available in hPSCreg, including for example human leukocyte antigen (HLA) and short tandem repeats (STR) data.

## IMPLEMENTATION

The hPSCreg web application's architecture can best be described as a four-tier system, consisting of a front-end application, i.e. user interface (UI), a middleware server-side application, a large set of specialized web-services and data stores. We follow the micro-services architecture pattern, for maximum flexibility, helping hPSCreg to scale well in the future. The front-end application, implemented using cutting edge HTML5, CSS3 and JavaScript technologies, communicates with a PHP-based middleware application, running on an Apache web server. The middleware securely handles the communication between UI and a variety of specialized Java-based micro web-services. These micro web-services process, send and retrieve data from the data stores. Data is simultaneously stored in a MySQL database management system and Elasticsearch. MySQL databases, act as the main data storage, while Elasticsearch enables a Google-like search experience. LDAP is used for authorizing users and managing groups.

The Registry has implemented a partnership with the European bank of induced pluripotent stem cells (EBiSC) and is used by this bank as a depositors’ portal for the registration of iPSC-lines for deposition in EBiSC. EBiSC is using hPSCreg's registration facility and nomenclature system to achieve standardized data provision and unambiguous naming of all lines. In addition, hPSCreg provides, through an API with the European Bioinformatics Institute (EBI), a BioSamples ID to each line registered for EBiSC. The BS-ID provides a unique identifier for aggregation of any sample related to the cell line, for example the donor, RNA, differentiated cells, lots and batches derived from the line, as well as relationships between samples such as ‘derived from’. Hence the implementation of a standard registration tool, a unique standard name and a BS-ID provides data traceability between different banks and institutions, and comparability down to the sample and lot level.

## DISCUSSION

The human pluripotent stem cell registry provides comprehensive and cell line specific information on human PSC-lines around the world. In contrast, most other registries are restricted to banks or to specific uses of lines such as the restriction to lines eligible for funding by NIH funds in the NIH registry (http://grants.nih.gov/stem_cells/registry/current.htm), or restricted to national cell lines, for example in Korean PSC Registry (11). Federated systems, such as eagle-i (12) provide links to different resources harboring PSC lines, but the information provided is not as standardized and comprehensive as what can be provided in a centralized database such as hPSCreg. Hence, hPSCreg is unique as a global registry providing comprehensive scientific and ethical information on human PSC lines. The task of defining minimal standards for human PSC line characterization is thus one of the key elements of hPSCreg and has been addressed via standard nomenclature and minimal PSC criteria. Moreover, hPSCreg is the only registry, which centrally delivers information on lines from multiple providers and banks facilitating effective use of the available resources. One of the key requirements for facilitating comparative evaluation over so many and diverse repositories are unique identifiers and standard criteria for registration and assessment - despite a large variety of experimental procedures used. We expect that in the ongoing consolidation process of the field, standards and protocols will be established and eventually dominate especially sensitive areas such as application of human PSC for clinical or drug testing applications. These areas require adherence to standards and guidelines defined also by regulatory agencies. The registry will adjust its registration formats accordingly and we expect to stratify the required fields further, for example to accommodate and validate human PSC lines suitable for clinical application.

In addition, the regulatory landscape was and still is characterized by diverse ethical conceptions on hPSC derivation and usage, which continuously requires transparency about the cell's origins, compliance with ethical standards and information about availability of the cells to researchers (13). The registry supports harmonizing the standards for obtaining informed consent for tissue donation and use, for example by codifying ethics information. A first step is the ethics registration form within hPSCreg's registration tool. Similarly, the development of suitable ontologies for information enrichment is a future task. While ontology and standardized terms are already being used in hPSCreg, many areas are not yet covered. Collaborative efforts for example between eagle-i and hPSCreg on the development of ontologies, informed consent codification and clinical data standards will be useful to enhance resource usage, comparability and applicability of cell lines.

One increasingly important application of human PSC is their use in drug efficacy studies, based on genetically stratified cohorts of individualized cell lines. This requires access to genetic data from the cells and is currently handled via data access committees. This elaborate process is prohibitive for the design of these kinds of studies aiming to use cells from different sources. Hence, there is a need to develop specific access protocols for potentially sensitive genetic data, which adheres to the standards established by the Global Alliance for Genomics and Health (http://genomicsandhealth.org).

## CONCLUSION AND OUTLOOK

hPSCreg was established in 2007 as the European human embryonic stem cell registry and is supported by the European Commission. Its initial goal to provide transparency to the field of stem cell research and to establish ethical standards has been extended to cover all fields of human pluripotent stem cell research. The registry now provides comprehensive scientific and ethical information on all hPSC lines and provides these to scientists, regulators, lawmakers and the general public. Future implementations will include integration of standards and validation tools for clinical grade human PSC, the registration of clinical trials using stem cells as well as the provision of APIs for programmatic access to hPSCreg's data and registry. An option for downloading certain parts of hPSCreg is currently tested.The registry will be sustained through further European and international funding, which will allow it to gradually develop additional tools, include more focused information on cell types derived from human PSC, disease-specific lines and implementation of feedback as well as customized data display and documentation tools.

## References

[1] Thomson J.A., Itskovitz-Eldor J., Shapiro S.S., Waknitz M.A., Swiergiel J.J., Marshall V.S., Jones J.M. (1998). Embryonic stem cell lines derived from human blastocysts. Science.

[2] Takahashi K., Tanabe K., Ohnuki M., Narita M., Ichisaka T., Tomoda K., Yamanaka S. (2007). Induction of pluripotent stem cells from adult human fibroblasts by defined factors. Cell.

[3] Borstlap J., Kurtz A., Stacey G., Elstner A., Damaschun A., Arán B., Gerlach J.C., Izpisúa J.C., Veiga A. (2008). Development of a European human embryonic stem cell registry. Regen. Med..

[4] Borstlap J., Stacey G., Kurtz A., Elstner A., Damaschun A., Arán B., Veiga A. (2008). First evaluation of the European hESCreg. Nat. Biotechnol..

[5] Gostev M., Faulconbridge A., Brandizi M., Fernandez-Banet J., Sarkans U., Brazma A., Parkinson H. (2012). The BioSample Database (BioSD) at the European Bioinformatics Institute. Nucleic Acids Res..

[6] Luong M.X., Auerbach J., Crook J.M., Daheron L., Hei D., Lomax G., Loring J.F., Ludwig T., Schlaeger T.M., Smith K.P. (2011). A call for standardized naming and reporting of human ESC and iPSC lines. Cell Stem Cell.

[7] Stachelscheid H., Seltmann S., Lekschas F., Fontaine J.F., Mah N., Neves M., Andrade-Navarro M.A., Leser U., Kurtz A. (2014). CellFinder: a cell data repository. Nucleic Acids Res..

[8] Seltmann S., Stachelscheid H., Damaschun A., Jansen L., Lekschas F., Fontaine J.F., Nguyen-Dobinsky N.N., Leser U., Kurtz A. (2013). CELDA – an ontology for the comprehensive representation of cells in complex systems. BMC Bioinformatics.

[9] Declarations of the Commission (Framework Programme) (2012). Information from european union institutions, bodies, offices and agencies european commission. Off. J. Eur. Union.

[10] Isasi R., Andrews P.W., Baltz J.M., Bredenoord A.L., Burton P., Chiu I.M., Hull S.C., Jung J.W., Kurtz A., Lomax G. (2014). Identifiability and privacy in pluripotent stem cell research. Cell Stem Cell..

[11] Lee J.Y., Lee D.Y., Choi Y.S., Lee K.J., Kim Y.O. (2011). Registration of human embryonic stem cell lines: Korea, 2010. Osong. Public Health Res. Perspect..

[12] Vasilevsky N., Johnson T., Corday K., Torniai C., Brush M., Segerdell E., Wilson M., Shaffer C., Robinson D., Haendel M. (2012). Research resources: curating the new eagle-i discovery system. Database (Oxford).

[13] Kurtz A., Stacey G., Kidane L., Seriola A., Stachelscheid H., Veiga A. (2014). Regulatory insight into the European human pluripotent stem cell registry. Stem Cells Dev..

